# Selective extended dissection for pancreaticoduodenectomy is associated with better survival in pancreatic cancer patients: retrospective cohort study

**DOI:** 10.1097/JS9.0000000000000437

**Published:** 2023-05-16

**Authors:** Xiaofan Guo, Yuning Song, Peijun Xu, Wenbo Zhu, Hongwei Wang, Yucheng Zhou, Chongbiao Huang, Jihui Hao, Song Gao

**Affiliations:** a Department of Pancreatic Cancer, Tianjin Medical University Cancer Institute and Hospital, National Clinical Research Center for Cancer, Key Laboratory of Cancer Prevention and Therapy, Tianjin; b Department of General Surgery, Zhejiang Provincial People’s Hospital, Wenzhou Medical University, 158 Shangtang Road, Hangzhou, Zhejiang Province, China

**Keywords:** extended dissection, pancreatic cancer, perineural invasion, whipple procedure

## Abstract

Pancreatic ductal adenocarcinoma is a highly malignant tumor with relatively poor survival. Surgery is the first choice for treating patients with early pancreatic cancer. However, the surgical approach and the extent of resection for patients with pancreatic cancer are currently controversial.

**Methods:**

The authors optimized the procedure of standard pancreaticoduodenectomy to selective extended dissection (SED), which is based on the extrapancreatic nerve plexus potentially invaded by the tumor. The authors retrospectively analyzed the clinicopathological data of patients with pancreatic adenocarcinoma who underwent radical surgery in our center from 2011 to 2020. Patients who underwent standard dissection (SD) were matched 2:1 to those who underwent SED using propensity score matching. The log-rank test and Cox regression model were used to analyze survival data. In addition, statistical analyses were performed for the perioperative complications, postoperative pathology, and recurrence pattern.

**Results:**

A total of 520 patients were included in the analysis. Among patients with extrapancreatic perineural invasion (EPNI), disease-free survival was significantly longer in those who received SED than in those who received SD (14.5 months vs. 10 months, *P*<0.05). The incidence of metastasis in No. 9 and No. 14 lymph nodes was significantly higher in patients with EPNI. In addition, there was no significant difference in the incidence rate of perioperative complications between the two surgical procedures.

**Conclusion:**

Compared with SD, SED exhibits a significant prognostic benefit for patients with EPNI. The SED procedure aiming at specific nerve plexus dissection displayed particular efficacy and safety in resectable pancreatic ductal adenocarcinoma patients.

## Introduction

Highlights

- Selective extended dissection (SED) is a novel surgical procedure, which is based on the extrapancreatic nerve plexus potentially invaded by the tumor in a patient with pancreatic head cancer.
- SED can significantly improve the disease-free survival of patients with extrapancreatic perineural invasion.
- SED can reduce the possibility of postoperative regional recurrence.
- The positive rate of No. 9 and No. 14 lymph nodes was higher in patients with extrapancreatic perineural invasion, and routinely extended dissection was recommended.

Pancreatic ductal adenocarcinoma (PDAC) is one of the most malignant tumors which had become the third leading cause of death among cancers in 2022^1^. Often for patients with regional lesions, surgery is the only potential curative option, even though early recurrence after radical resection is extremely high. The importance of extended dissection in patients with resectable pancreatic cancer still remained undefined. Several randomized controlled trials, as well as some retrospective studies have proved that comparing with standard dissection (SD), patients underwent extended dissection exhibited no significant survival benefit^2–5^. In addition, the prognosis of patients with resectable pancreatic cancer can be improved by total neoadjuvant therapy, including preoperative radio-chemotherapy, radical dissection, and postoperative radio-chemotherapy^6,7^. This treatment concept may impair the importance of radical surgery in multidisciplinary treatment, while emphasize the role of perioperative chemoradiotherapy. Nevertheless, the procedure of operation and extent of resection are still the focused area of research. On the one hand, radical dissection is the most efficient treatment to eliminate the primary tumor burden, so that patients’ symptoms such as jaundice and pain, could be relieved or eliminated. On the other hand, postoperative pathology can provide a complete pathological stage and molecular typing, which can provide the foundation for subsequent treatment.

For the standard operation in patients with pancreatic head cancer, the whipple procedure, the optimal extent of resection had been still controversial^8^. The results of several clinical trials showed that extended dissection did not significantly influence the long-term prognosis in patients received radical dissection. However, the surgical procedure and extent of resection in clinical trials is hardly reflective of a real-world situation. Firstly, a comprehensive evaluation based on the general state, physical state, preoperative laboratory value, and imaging examination are needed to determine whether patients should receive extended dissection. In addition, the extent of resection should be adapted according to the anatomical subsites of the tumors and their biological characteristics^9^. Therefore, for patients with pancreatic head cancer, the extent of resection cannot be a single unified model. In summary, we aimed to identify patients with resectable pancreatic cancer who might benefit from extended surgery.

Extrapancreatic nerve invasion (EPNI) is a malignant biologicals behavior of PDAC, as well as a risk factor of prognosis for patients^10,11^. Our previous studies have shown that preoperative enhanced-contrast CT yielded a high diagnosis sensitivity and specificity on EPNI. In addition, tumors that arise at different anatomic sites of the pancreas potentially invaded different nerve plexus^12^. In this study, we proposed a novel surgical procedure of selective extended dissection (SED) focusing on dissecting the nerve plexus selectively based on the nerve plexus potentially invaded by tumor. On the one hand, the ‘selective’ of SED refers to the selection of the region of nerve plexus. According to the classification of preoperative imaging perineural invasion, the nerve plexus with potential invasion should be dissected. The ‘selective’ of SED, on the other hand, refers to the selection of patients most likely to derive benefit from extended dissection, according to EPNI. Extended dissection is just performed in patients with EPNI, as shown by preoperative imaging examination, while SD is performed in patients without EPNI. In order to perform an accurate and comprehensive dissection of the nerve plexus, different arterial approaches were combined with extended dissection. On the basis of the normalization of the surgical procedure, the patients who received SED underwent long-termed follow-up to evaluate the efficacy and safety of this procedure.

## Method

### Study population and data collection

We reviewed the medical records of patients who were admitted to our center from January 2011 to December 2019 from the medical database of our center. The inclusion criteria are as follows: Patients of any age diagnosed as resectable pancreatic cancer were pathologically diagnosed as PDAC after surgery. Patients received radical surgery and had complete photos or videos of the operation. Patients had no history of cancer including pancreatic cancer. Exclusion criteria: The operation records and related photo or video data could not reflect the surgical approach and the extent of resection or surgical rule violation. Patients with incomplete clinical, pathological, imaging, and follow-up information. An unresectable condition or metastasis was found during surgery. The diagnosis of resectable pancreatic cancer and postoperative chemotherapy regimen were based on the guidelines of National Comprehensive Cancer Network (NCCN) guidelines^13^. The postoperative specimens were evaluated according to AJCC criteria (8th edition)^14^. This retrospective study met the ethical guidelines of the Declaration of Helsinki and was approved by the Ethics Committee of our center (bc2023010).

### Surgical procedure

According to the extent of nerve invasion in patients with pancreatic cancer and the development of the pancreas during embryogenesis, we classified the patients into three types and determined the scope of resection. For type I EPNI, the tumor is located in the dorsal pancreatic head or pancreatic neck, which tends to invade extrapancreatic nerve plexus 1 (PLX1) surrounding the celiac trunk (CT). Type II refers to the tumor located in the ventral head of the pancreas (uncinate process of the pancreas), and the tumor tends to be invaded in the extrapancreatic nerve plexus 2 (PLX2) surrounding the superior mesenteric artery (SMA). Type III is a pancreatic tumor prone to comprehensively invade PLX1 and PLX2. Different surgical techniques were developed for different types, which are as follows.

**Figure 1:**
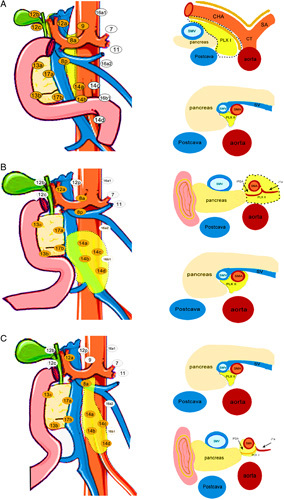
The diagram for the extent of extrapancreatic nerve plexus and lymph nodes of selective extended dissection (SED): A. type I extrapancreatic perineural invasion (EPNI), B. type II EPNI and C. type III EPNI. The yellow region was the nerve plexus should be dissected and orange dots refer to the lymph nodes. CHA, common hepatic artery; CT, celiac trunk; PLX I, extrapancreatic nerve plexus I; PLX II, extrapancreatic nerve plexus II; SA, spleen artery; SV, spleen vein; SMA, superior mesenteric artery; SMV, superior mesenteric vein.

For the patients with type I EPNI, we focused on an extended dissection of PLX1 during surgery. The detailed extent of nerve fibers, lymph nodes, and soft tissue are shown in Figure 1A and Supplementary Figure 1a, Supplemental Digital Content 2, http://links.lww.com/JS9/A503. Following the Cattell-Braasch maneuver, the gastrocolic ligament and the greater omentum were dissected until the head of the pancreas is fully exposed and mobilized. Soft tissues along the inferior border of the pancreas was released to expose the anterior wall of the superior mesenteric vein (SMV) and portal vein (PV). Then, soft tissue along the left side of the SMV were dissected to exposed SMA. After the transection of the pancreatic neck, the mesopancreas along the right lateral of the SMA were separated, and the arterial segments between the root of the SMA and the horizontal at the inferior border of the pancreas were exposed. Then, PLX1 was exposed between SMV and SMA. Lymph nodes and nerve plexus bearing soft tissues along the celiac axis (CA), SMA, and SMV/PV was completely dissected (TRIANGLE operation). And then No. 8a (the CHA), No. 9 (the CA), and No. 12 (portal hilum) lymph nodes were removed en bloc, and the arterial adventitia of CHA, CA (left side), and SMA (root part of the artery) were resected.

For the situation of type II, the tumor was closely related to the PLX2. A left-posterior artery approach was performed. The Treitz ligament was dissected to expose the SMA. After the proximal jejunum was dissected and overturns to the right, the SMA was rotated counterclockwise. The first jejunal artery (J1A) was isolated and ligated at its root either on the SMA or on the common trunk with the inferior pancreaticoduodenal artery (IPDA). Then, the mesojejunum around the IPDA and J1A and the left hemicircle nerve plexus of SMA were dissected. Through a Cattell-Braasch maneuver performed^15^, the root of SMA was isolated above the left renal vein. After that, SMV was completely isolated from the right of the SMA, and the right hemicircle nerve plexus of SMA was dissected. At this point, the PLX2 and No. 14 lymph nodes surrounding the SMA have been dissected.

Patients with type III EPNI were considered concurrent invasion of the tumor to both PLX1 and PLX2. The extent of resection included lymph nodes, nerve plexus and soft tissue along the entire circumference of SMA as well as the Heidelberg Triangle. In addition to this, CHA and right side of SMA, were skeletonized.

In routine extended dissection (RED), on the basis of SD, the CHA lymph nodes (No. 8), CT lymph nodes (No. 9), SMA lymph nodes (No. 14), and para-aortic lymph nodes between CT and inferior mesenteric artery (No.16a2, No.16b1) were dissected. All the soft tissues around the hepatoduodenal ligament were completely dissected and skeletonized. The nerve plexus or ganglion on the right hemicircle of the celiac trunk and the peripheral nerve plexus along the entire circumference of SMA were dissected^2^.

### Clinicopathologic data

Clinical, pathological, and imaging information were entered into the pancreatic cancer database by two independent researchers. Surgical videos and photos were collected by researchers who were not involved in the operation and uploaded to the database of pancreatic cancer. Follow-up information was uploaded to the database after regular telephone follow-up by the follow-up team of our center. The above patients’ clinical, imaging, and pathological information were collected from the pancreatic cancer database. Contrast-enhanced CT evaluated the EPNI of all the patients. The pathological data of the patients were mainly concerned with the EPNI. The diagnosis and subtypes of imaging PNI was based on the criteria proposed in our previous studies. Pathologically, the diagnostic criterion of EPNI is a nerve plexus invaded by tumor tissue was no surrounding benign pancreatic tissue (islet of Langerhans, pancreatic acini, or benign pancreatic ductles)^16^. In addition, the dissected nerve plexus during surgery was pathologically examined in patients who received SED. In addition, the shortest distance between the tumor boundary and the adventitia of the CT and SMA on enhanced-contrast CT was less than 6.5 mm as the diagnostic criteria for imaging PNI^12^.

### Study design and statistical methods

The propensity score matching method matched patients undergoing SED and patients undergoing SD according to sex, age, 8th AJCC stage, pathological grade, and serum CA19-9 level in a 1:2 ratio. The nearest matching method was used, and the caliper value was set at 0.05. In the matched cohort, the standardized mean difference of each index was less than 0.1, as shown in Supplementary Figure 2, Supplemental Digital Content 2, http://links.lww.com/JS9/A503

The measurement data were described by mean, SD, median, and interquartile range, while count data was described as the number of cases and percentage. Statistical analysis was performed on the matched cohort, and the *t*-test or Mann–Whitney *U* test was used for measurement data. Categorical data were analyzed by the *χ* ^2^-test or Fisher’s exact test. All statistical analyses were performed R and R Studio software (version 1.4.1106.0). The applied packages include tableone, matching, survey, reshape2, ggplot2, survival, and survfit. In this study, *P*<0.05 was considered statistically significant. This work has been reported in line with strengthening the reporting of cohort studies in surgery (STROCSS) criteria^17^, Supplemental Digital Content 1, http://links.lww.com/JS9/A502.

## Result

### Comparison of baseline information between the two groups

**Figure 2:**
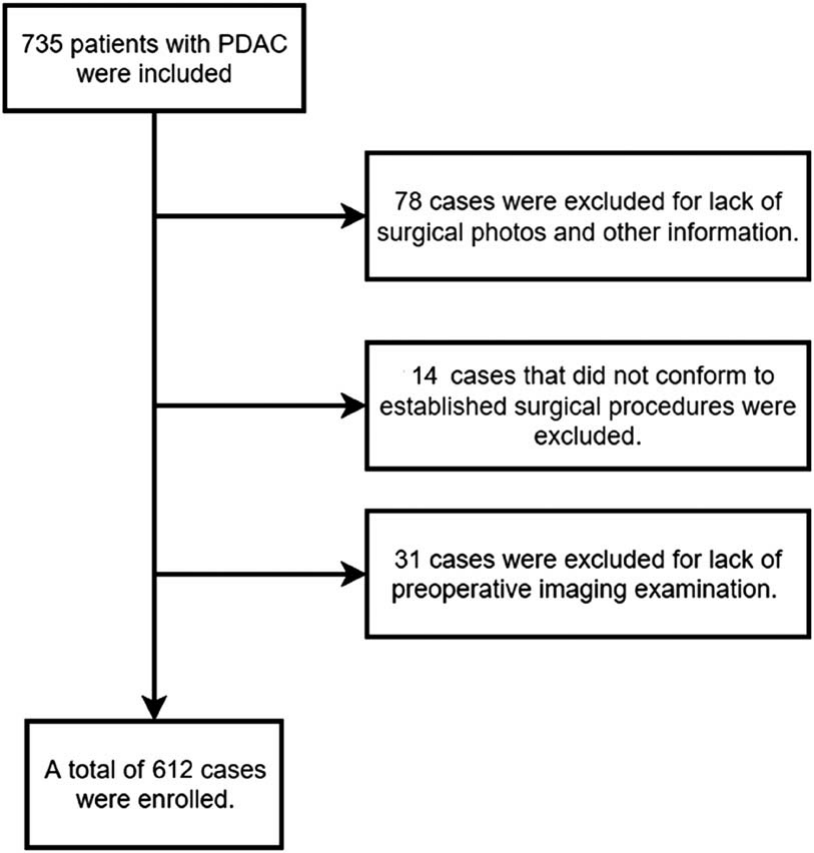
Flow chart of patient enrollment and exclusion.

According to the established criteria, 735 cases were included, 118 cases were excluded, and 612 patients were finally included, as shown in Figure 2. Patients were followed through the death date or the last follow-up date (31 December 2022), and the median follow-up time overall was 46 months (interquartile range, 40–60 months). We compared the clinicopathological data between the SD group and the SED group. Significant differences were noted between the two groups in terms of AJCC stage, pathological grade, vascular invasion rate, and R0 resection rate, as well as the number of cases between the two groups (shown in Supplementary Table 1, Supplemental Digital Content 2, http://links.lww.com/JS9/A503). For the cohort after matching, differences between the two groups of patients in the above clinicopathological characteristics were eliminated, as shown in Table 1. The matched cohort consisted of 243 patients, 162 cases in the SD group, and 81 cases in the SED group.

**Table 1:** Comparison of basic information between SD and SED group in matched cohort.

	SD	SED	*P*
	162	81	
Sex (%)			
Female	76 (46.9)	40 (49.4)	0.820
Male	86 (53.1)	41 (50.6)	
Age (%)			
≤60	71 (43.8)	36 (44.4)	>0.999
>60	91 (56.2)	45 (55.6)	
Tumor size (%)			
<=4	108 (66.7)	59 (72.8)	0.406
>4	54 (33.3)	22 (27.2)	
*P* stage (%)			
I/IIa	54 (33.3)	22 (27.2)	0.406
IIb/III	108 (66.7)	59 (72.8)	
Positive LN ratio (%)			
≤0.02	76 (46.9)	35 (43.2)	0.682
>0.02	86 (53.1)	46 (56.8)	
Soft tissue invasion (%)			
Negative	36 (22.2)	18 (22.2)	>0.999
Positive	126 (77.8)	63 (77.8)	
Vessel invasion (%)			
Negative	137 (84.6)	69 (85.2)	>0.999
Positive	25 (15.4)	12 (14.8)	
Differentiation (%)			
Poor	30 (18.5)	16 (19.8)	0.954
Well	132 (81.5)	65 (80.2)	
CA19-9 (%)			
≤172.8 U/ml	74 (45.7)	40 (49.4)	0.683
>172.8 U/ml	88 (54.3)	41 (50.6)	
CEA (%)			
≤3.54 ng/ml	85 (52.5)	41 (50.6)	0.892
>3.54 ng/ml	77 (47.5)	40 (49.4)	
Nerve invasion (%)			
Negative	42 (25.9)	21 (25.9)	>0.999
Positive	120 (74.1)	60 (74.1)	
IPNI (%)			
iPNI (-)	67 (41.4)	35 (43.2)	0.890
iPNI (+)	95 (58.6)	46 (56.8)	
Postoperative chemotherapy (%)			
No	46 (28.4)	23 (28.4)	>0.999
Yes	116 (71.6)	58 (71.6)	
Curability (%)			
R0	94 (58.0)	47 (58.0)	>0.999
R1	68 (42.0)	34 (42.0)	

CA19-9, carbohydrate antigen 19-9; CEA, carcinoembryonic antigen; iPNI, imaging Perineural Invasion; LN, lymph node; SD, Standard Dissection; SED, Selective Extended dissection.

### Survival analysis and subgroup analysis

**Figure 3:**
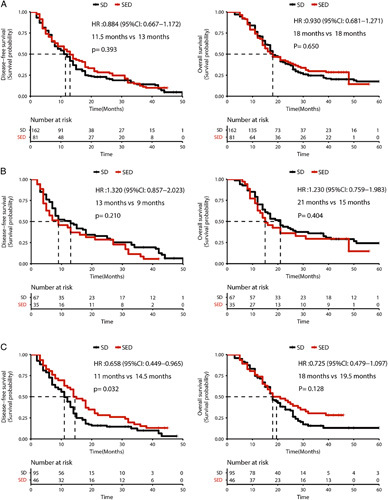
K-M curve for patients underwent selective extended dissection (SED) and standard dissection (SD): A. in the total cohort, B: in patients without extrapancreatic perineural invasion (EPNI) and C: in patients with EPNI.

In the matched cohort, patients had no significant difference in DFS and OS between the SED group and the SD group (DFS: *P*=0.393; OS: *P*=0.650), as shown in Figure 3A. In the subgroup of patients without EPNI indicated by preoperative enhanced-contrast CT, no significant difference in DFS and OS was indicated between the two groups (DFS: *P*=0.210; OS: *P*=0.404), as shown in Figure 3B. However, in the subgroup of patients with EPNI, SED group had a significantly better DFS than that of SD group (median DFS: 14.5 months vs. 11 months, *P*=0.032), while there was no significant difference in OS between the two groups, as shown in Figure 3C. Thus, patients with EPNI, the SED can significantly prolong the DFS of patients. However, this effect was not significant in patients without EPNI.

Cox regression analysis for the matched cohort showed that tumors larger than 4 cm, AJCC stages IIb to III, and R1 resection were independent risk factors for DFS and OS, as shown in Table 2. In addition, tumor size larger than 4 cm, lymph node positive rate greater than 0.02, and R1 resection are independent risk factors for OS, as shown in Table 3. Although in Cox hazard regression models, SED was not a protective factor for the prognosis of patients with pancreatic cancer. These results demonstrate that SED is of great significance for patients with potential EPNI, which is significantly able to improve the DFS of patients. However, in patients without EPNI, the improvement of survival for extended dissection was not significant.

**Table 2:** Univariate and multivariate cox regression model for DFS in matched cohort.

	Univariate analysis		Multivariate analysis	
	HR (95% CI for HR)	*P*	HR (95% CI for HR)	*P*
Sex				
Female/Male	0.934 (0.715–1.219)	0.615		
Age				
≤60/>60	0.874 (0.668–1.140)	0.317		
Tumor size				
≤4/4 cm	1.632 (1.228–2.170)	<0.001	2.050 (1.506–2.791)	<0.001
8th AJCC stage				
I, IIa/IIb,III	1.406 (1.055–1.874)	0.020	1.892 (1.343-2.665)	<0.001
Positive LN ratio				
≤0.02/0.02	1.462 (1.071–1.997)	0.017	1.264 (0.899–1.777)	0.178
Soft tissue invasion				
Negative/Positive	1.033 (0.752–1.420)	0.840		
Vessel invasion				
Negative/Positive	1.021 (0.698–1.493)	0.916		
Differentiation				
Poor/Well	0.816 (0.580–1.147)	0.241		
CA19-9				
≤200/>200 U/ml	1.297 (0.994–1.694)	0.056		
CEA				
≤3.5/>3.5 ng/ml	0.973 (0.746–1.270)	0.842		
iPNI				
Negative/Positive	1.048 (0.800–1.374)	0.732		
Postoperative chemotherapy				
No/Yes	0.796 (0.594–1.065)	0.125		
Curability				
R0/R1	1.414 (1.081–1.850)	0.012	1.739 (1.311–2.305)	<0.001

AJCC, American Joint Committee on Cancer Staging; CA19-9, carbohydrate antigen 19-9; CEA, carcinoembryonic antigen; HR, hazard ratio; iPNI, imaging perineural invasion; LN, lymph node.

**Table 3:** Univariate and multivariate analysis of OS in matched cochort.

	Univariate analysis			Mutivariate analysis	
	HR (95% CI for HR)	*P*		HR (95% CI for HR)	*P*
Sex					
Female/Male	0.789 (0.591–1.055)		0.110		
Age					
≤60/>60	0.787 (0.589–1.052)		0.105		
Tumor Size					
≤4/4 cm	1.780 (1.317–2.406)		<0.001	1.859 (1.373–2.517)	<0.001
p stage					
I, IIa/IIb,III	1.370 (1.000–1.877)		0.050		
LN ratio					
≤0.02/0.02	1.589 (1.145–2.207)		0.006	1.775 (1.271–2.478)	0.001
Soft tissue invasion					
Negative/Positive	1.150 (0.810–1.634)		0.434		
Vessel invasion					
Negative/Positive	1.296 (0.880–1.908)		0.190		
Differentiation					
Poor/Well	0.775 (0.539–1.113)		0.168		
CA19-9					
≤200/>200 U/ml	1.234 (0.923–1.652)		0.156		
CEA					
≤3.5/>3.5 ng/ml	0.807 (0.603–1.081)		0.150		
EPNI					
Negative/Positive	1.155 (0.857–1.556)		0.343		
Postoperative chemotherapy					
No/Yes	0.772 (0.564–1.056)		0.105		
Curability					
R0/R1	1.441 (1.077–1.928)		0.014	1.561 (1.162–2.098)	0.003
Surgical approach					
SD/SED	0.931 (0.681–1.271)		0.650		

AJCC, American Joint Committee on Cancer Staging; CA19-9, carbohydrate antigen 19-9; CEA, carcinoembryonic antigen; EPNI, extrapancreatic perineural invasion; HR, hazard ratio; LN, lymph node.

### Postoperative pathology analysis

**Figure 4:**
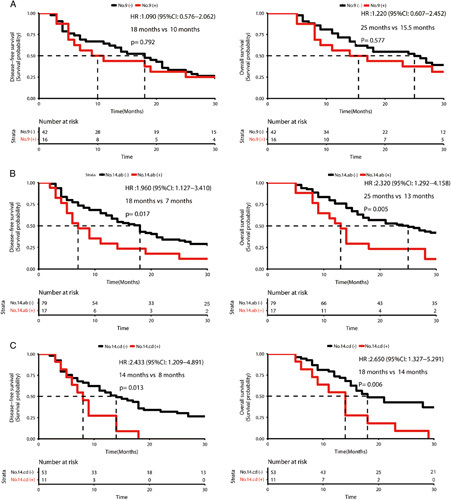
Comparison of K-M curve between patients with and without regional lymph nodes metastasis: A. No. 9 lymph nodes, B. No. 14a or No. 14b lymph nodes and C. No. 14c and No. 14d lymph nodes.

According to the statistical analysis of postoperative pathology in the matched cohort, it can be concluded that EPNI is highly correlated with metastasis of No. 9 and No. 14 lymph nodes. In the EPNI positive group, the positive rate of lymph nodes in No. 14c or No. 14d (28.6%) was significantly higher than that in the EPNI negative group (3.4%). It is worth nothing that the positive rate of No. 9 lymph nodes (38.9%) was also significantly higher than that in the EPNI negative group (9.1%), as shown in Table 4. In addition, the pathological examination of the surgical specimens for the PLX1 and PLX2 further confirmed the diagnostic value of contrast-enhanced CT in pathological EPNI. There was no significant difference in prognosis between patients with positive and negative No. 9 lymph nodes, as shown in Figure 4A. The patients with negative No. 14 lymph nodes showed a markedly better prognosis than those of the negative patients, as shown in Figures 4B and C.

**Table 4:** Comparison of lymph node metastasis between EPNI positive and negative patients after operation.

	Overall	EPNI (-)	EPNI (+)	*P*
	96	40	56	
Positive LN (%)				
<=3	60 (62.5)	28 (70.0)	32 (57.1)	0.285
>3	36 (37.5)	12 (30.0)	24 (42.9)	
LN dissection (%)				
<=24	54 (56.2)	26 (65.0)	28 (50.0)	0.211
>24	42 (43.8)	14 (35.0)	28 (50.0)	
Type (%)				
I	32 (33.3)	11 (27.5)	21 (37.5)	0.545
II	38 (39.6)	18 (45.0)	20 (35.7)	
III	26 (27.1)	11 (27.5)	15 (26.8)	
PLX1 (%)	28 (56.0)	2 (14.3)	26 (72.2)	0.001
PLX2 (%)	30 (54.5)	4 (20.0)	26 (74.3)	<0.001
EPNI (%)	47 (49.0)	6 (15.0)	41 (73.2)	<0.001
No. 14.ab (%)	17 (17.7)	4 (10.0)	13 (23.2)	0.161
No. 14.cd (%)	11 (17.2)	1 (3.4)	10 (28.6)	0.020
No. 9 (%)	16 (27.6)	2 (9.1)	14 (38.9)	0.031
No. 8a (%)	39 (40.6)	15 (37.5)	24 (42.9)	0.752
No.8p (%)	22 (22.9)	9 (22.5)	13 (23.2)	>0.999
No.7 (%)	7 (7.3)	3 (7.5)	4 (7.1)	>0.999
No. 5 (%)	9 (9.4)	4 (10.0)	5 (8.9)	>0.999
No. 6 (%)	6 (6.2)	4 (10.0)	2 (3.6)	0.392
No.13 (%)	46 (47.9)	19 (47.5)	27 (48.2)	>0.999
No.17 (%)	38 (39.6)	16 (40.0)	22 (39.3)	>0.999

EPNI, extrapancreatic perineural invasion; LN, lymph node; PLX1, extrapancreatic perineural invasion 1; PLX2, extrapancreatic perineural invasion 2.

### Postoperative complications and recurrence pattern

There was no significant difference in the incidence of postoperative pancreatic fistula, biliary fistula, delayed gastric emptying, gastrointestinal fistula, abdominal infection, and postoperative hemorrhage between the two groups. However, the incidence of postoperative diarrhea in patients undergoing SED (23.5%) was higher than that in patients undergoing SD (8.6%). In addition, no significant differences were indicated in operation time, hospital stay, intraoperative blood loss, vascular reconstruction and combined organ resection between the two groups, as shown in Table 5. Comparing the postoperative recurrence pattern between the two groups, we found that patients who underwent SED (8.6%) had a lower peritoneal metastasis than those who underwent standard radical resection (21.6%). There were no significant differences in the incidence of stump recurrence, abdominal cavity metastasis, liver metastasis, lung metastasis, and bone metastasis, as shown in Table 6.

**Table 5:** Perioperative complications were compared between the two groups.

	Overall	SD	SED	*P*
	243	162	81	
Operation time (median [IQR])	291.92 [223.98, 334.18]	296.17 [231.13, 334.22]	285.86 [212.54, 333.19]	0.519
<=291.9	121 (49.8)	79 (48.8)	42 (51.9)	0.751
>291.9	122 (50.2)	83 (51.2)	39 (48.1)	
Intraoperative bleeding (median [IQR])	380.00 [270.00, 530.00]	380.00 [250.00, 517.50]	410.00 [280.00, 560.00]	0.171
<=380	122 (50.2)	84 (51.9)	38 (46.9)	0.555
>380	121 (49.8)	78 (48.1)	43 (53.1)	
Intraoperative transfusion (median [IQR])	0.00 [0.00, 500.00]	0.00 [0.00, 500.00]	0.00 [0.00, 500.00]	0.904
Combined devisceration (%)	13 ( 5.3)	9 (5.6)	4 (4.9)	
Vascular reconstruction (%)	21 ( 8.6)	15 (9.3)	6 (7.4)	
HLOS (mean (standard deviation))	20.15 (9.26)	20.43 (9.47)	19.59 (8.84)	0.509
<=19	125 (51.4)	81 (50.0)	44 (54.3)	0.618
>19	118 (48.6)	81 (50.0)	37 (45.7)	
Pancreatic fistula (%)				
No	149 (61.3)	95 (58.6)	54 (66.7)	0.393
Biochemical fistula	77 (31.7)	56 (34.6)	21 (25.9)	
Grade B	17 (7.0)	11 (6.8)	6 (7.4)	
Bile fistula (%)	58 (23.9)	36 (22.2)	22 (27.2)	0.489
Gastrointestinal fistula (%)	11 (4.5)	7 (4.3)	4 (4.9)	>0.999
DGE (%)	29 (11.9)	19 (11.7)	10 (12.3)	>0.999
Diarrhea (%)	33 (13.6)	14 (8.6)	19 (23.5)	0.003
Postoperative bleeding (%)	22 (9.1)	14 (8.6)	8 (9.9)	0.937

DGE, delayed gastric emptying; HLOS, hospital length of stay; IQR, interquartile range; SD, standard dissection; SED, selective extended dissection.

**Table 6:** Comparison of postoperative recurrence pattern between SD and SED.

	Overall	SD	SED	*P*
	243	162	81	
Residual pancreas (%)	5 (2.1)	5 (3.1)	0 (0.0)	0.263
Liver metastasis (%)	89 (36.6)	64 (39.5)	25 (30.9)	0.239
Lung metastasis (%)	32 (13.2)	22 (13.6)	10 (12.3)	0.947
Bone metastasis (%)	7 (2.9)	2 (1.2)	5 (6.2)	0.078
Peritoneal metastasis (%)	42 (17.3)	35 (21.6)	7 (8.6)	0.019
Retroperitoneal lymph nodes metastasis (%)	70 (28.8)	53 (32.7)	17 (21.0)	0.080

SD, standard dissection; SED, selective extended dissection.

### Comparison between SED and RED

We compared survival between patients who underwent SED and RED. In the original cohort, the proportion of patients receiving postoperative chemotherapy was significantly higher in the SED group than in the RED group (67.7 vs. 29.3%, *P*<0.001). There was no significant difference in other clinicopathological data between the two groups, as shown in supplementary Table 2, Supplemental Digital Content 2, http://links.lww.com/JS9/A503. In addition, SED had a significantly better DFS than those in the RED group (14 months vs. 9 months), while there was no significant difference in OS between the two groups (21 months vs. 18 months), as shown in supplementary Figure 3A, Supplemental Digital Content 2, http://links.lww.com/JS9/A503. Among patients with EPNI, DFS, and OS in SED group were significantly better than those in the SD group, as shown in supplementary Figure 3B, Supplemental Digital Content 2, http://links.lww.com/JS9/A503. However, among patients without EPNI, there was no significant difference in DFS and OS between the two groups, as shown in supplementary Figure 3C, Supplemental Digital Content 2, http://links.lww.com/JS9/A503. In addition, the operation time (median: 282.29 min vs. 306.88 min) and HLOS (19.43 days vs. 27.63 days) were significantly higher in the RED group, as shown in supplementary Table 3, Supplemental Digital Content 2, http://links.lww.com/JS9/A503. The incidence of DEG after surgery as well as diarrhea was significantly higher in the RED group than in the SED group, as shown in supplementary Table 3, Supplemental Digital Content 2, http://links.lww.com/JS9/A503. The RED group had a significantly higher incidence of postoperative liver metastases than those in the SED group, as shown in supplementary Table 4, Supplemental Digital Content 2, http://links.lww.com/JS9/A503.

## Discussion

Previous studies have shown that EPNI is a risk factor for the prognosis of pancreatic cancer. EPNI is closely related to a variety of malignant biological behaviors, such as lymph node metastasis and liver metastasis^18,19^. The extent of surgical resection for patients with EPNI and the dissection of the extrapancreatic nerve plexus is still controversial. Inoue *et al*.^20^ proposed a surgical technique for aggressive pancreatic head tumors in which an en bloc resection of the nerve plexus was performed around the SMA on the right hemicircumference. Qian *et al*.^9^ also put forward a similar point of view and confirmed that No. 14 lymph node metastasis is likely to occur in ventral pancreatic cancer. This surgical technique can dissect the mesopancreas and soft tissue around SMA for the tumor located in the pancreatic uncinate process. However, this approach cannot be applied in this situation for malignant tumors located in the dorsal head and neck of the pancreas that are prone to invade CA/CHA and PLX1. Hackert et al. proposed the Heidelberg technique, which focuses on extended dissection of soft tissue with presumed tumor invasion within the PV/SMV, CA, and SMA boundary triangles^21^. This technique highlights the CA and SMA dissection in the right hemiperimetric region. However, there is no clear conclusion on whether the mesojejunum around J1A and IPDA needs to be dissected. Nagakawa *et al*. proposed a surgical resection method based on SMA’s peripheral nerve and fibrous tissue structure. They emphasized that preserving three regions without nerve and fibrous tissue branches can effectively reduce the incidence of postoperative serious adverse events while ensuring the R0 resection rate^22^. Previous studies about extended dissection of pancreaticoduodenectomy have always made uniform requirements for the scope of dissection. There is a lack of reasonable classification methods and a reasonable definition of the dissection scope for different types. Previous randomized controlled trials have concluded that there is no significant difference in OS benefit between the extended radical resection group and the standard radical resection group^2,4,5,23^. However, we believe that extended extrapancreatic nerve plexus dissection is beneficial for pancreatic cancer patients with EPNI. Therefore, we proposed the concept of SED. Compared with a merely ‘anatomical’ margin achieved by SD, a more radical ‘biological’ R0 margin is crucial for those PDAC patients with EPNI. On the one hand, PD with extended dissection on extrapancreatic nerve plexus can be benefit on patients diagnosed with imaging EPNI, whereas patients considered to have no EPNI before surgery should undergo SD to avoid a variety of postoperation complications. The selection, on the other hand, is to identify the extent of dissection. Depending on the type of imaging EPNI, the extrapancreatic nerve plexus that might be invaded by the tumor was accurately removed through SED technic; while for patients without imaging EPNI, SD was a more favorable approach. Our data have concluded that SED could improve the DFS of patients with EPNI, but barely show significance for patients without EPNI. Furthermore, SED has relatively lower retroperitoneal lymph node metastasis rates, residual pancreatic recurrence, and peritoneal seeding compared with SD. Therefore, we believe that expanded dissection with a focus on the nerve plexus can reduce the possibility of local and regional recurrence. The incidence of postoperative complications in the extended dissection group was similar to that in the SD group. Although the incidence of diarrhea in the extended dissection group was higher than in the SD group, most patients who received medical treatment had significant relief of diarrhea symptoms. Thus, SED has relatively favorable efficacy and safety.

Previous studies have shown that regional lymph node metastasis is a prognostic risk factor in patients with pancreatic cancer^24–26^. Current standards for lymph node dissection in standard radical resection of pancreatic cancer require No. 5, No. 6, No. 8a, No. 12b1, No. 12b2, 12C, No. 13a, No. 13b, right side of No. 14a, right side of 14b lymph node, No. 17a, and No.17b lymph nodes^27^. Qian *et al*.^9^ have confirmed that No. 14 lymph node metastasis is likely to occur in ventral pancreatic cancer and they recommended routine dissection of No. 14c, No. 14d lymph nodes in patients with malignancies of the pancreatic uncinate process. Our data suggested that the positive rate of No. 14c and No. 14d was significantly higher in patients with type II and III EPNI (PLX2 potentially invaded by the tumor). The patients with No. 14 lymph nodes metastasis exhibited a significantly poor prognosis. In addition, for patients with type I and III EPNI (PLX1 potentially invaded by the tumor), the positive rate of No. 9 lymph nodes was significantly higher than that in patients with negative EPNI. Therefore, routine dissection of No. 9 lymph nodes is recommended for patients with preoperatively considered PLX1 invaded, while dissection of No. 14 lymph nodes was performed in patients with PLX2 involvement. The SED approach may meet the requirement of selective extended lymphadenectomy including both No. 9 and No. 14 lymph nodes. On the one hand, SED was meaningful for the prolong of patients DFS; on the other hand, it would markedly improve the amount of collected lymph nodes so that a more accurate pathological staging could be performed.

Compared with SED, the incidence of delayed gastric emptying and diarrhea was significantly higher in the RED group. These are highly associated with excessive extrapancreatic nerve fiber dissection as well as the skeletonization of blood vessels. Postoperative adjuvant therapy was delayed or abandoned because of long-term nutritional disorders and poor physical status. These are the reasons why the proportion of patients in the RED group receiving adjuvant chemotherapy is significantly lower than that in the SED group. In addition, vascular injury caused by dissection of the perivascular soft tissue and adventitia may damage the local function of chemotherapy drugs. We believe that these may partly explain the worse prognosis of patients in the RED group. Therefore, even for resectable pancreatic cancer, timely and standardized postoperative adjuvant therapy is an important factor to improve the prognosis of patients. Although RED has a larger extent of dissection than SED, excessive dissection leads to poor physical status, which affects the postoperative treatment and is not conducive to the prognosis of patients. In conclusion, SED is a balance between SD and RED, which can accurately dissect the nerve and soft tissue potentially involved by the tumor, and also ensure the normal organ function of the patient to a great extent.

Regarding the R0 resection rate, there was no significant difference between patients in the SED group (42.0%) and those in the SD group (42.0%). Kaltenmeier et al. showed that the R1 resection rate was 14.4% in patients with pancreatic cancer, based on the resection margin cleared by more than 1 mm, and R1 resection margin was a risk factor for the prognosis^28^. Chang et al. showed that R1 resection rates were 51.5, 53.7, and 57.5%, respectively, based on the standard of 1 mm, 1.5 mm, and 2 mm, and a negative margin greater than 1.5 mm was a protective factor for the long-term prognosis of patients^29^. A clinical trial showed a 37.6% R1 resection rate, based on a resection margin more than 1 mm, after neoadjuvant chemotherapy in patients with borderline resectable pancreatic cancer^7^. Although, according to the NCCN guidelines, the standard of resection margin is greater than 1 mm, the distance greater than 2 mm as the standard of positive resection margin can effectively evaluate the prognosis of patients. Due to the high rate of early local recurrence around the resection margin and the discrepancy between pathological assessment and poor clinical outcomes, microscopic margin involvement is often underestimated^30^. Therefore, 2.5 mm was adopted as the criteria to evaluate the resection margin in our center while the R1 resection rate reached 42.0% in our cohort. Under this criteria, R1 resection is an independent risk factor for overall survival. This more stringent evaluation criteria cannot only reflect the biological characteristics of pancreatic cancer itself, but also prompt clinicians to carry out subsequent treatment to reduce the impact of R1 resection on the prognosis of patients.

This study systematically introduces the diagnostic indicators of EPNI, the prognostic value of iPNI, and the guiding significance of iPNI in selecting a resection range for pancreatic cancer. The limitation of this study is that it is a single-center, retrospective, and cohort study. This conclusion needs to be further verified by multicenter-controlled trials.

## Ethical approval

This study was approved by the Ethics Committee of Tianjin Medical University Cancer Institute and Hospital (bc2023010).

## Sources of funding

This work was supported by the National Key Research and Development Program of China(2021YFA1201100), the National Natural Science Foundation of China (grants 82072657).

## Author contribution

X.G.: the main designer and executor of the study and responsible for the overall design of the study. The writing of the manuscript and the revision of the manuscript. Y.S.: the principal investigator of the study and is responsible for the collection of data in the database. P.X.: one of the researchers involved in the collection of data from the database. W.Z.: one of the study researchers involved in the follow-up of case data. H.W.: one of the study researchers involved in the patient’s surgical procedures. Y.Z.: one of the study researchers involved in the patient’s surgical procedures. C.H.: one of the study designers involved in the overall design of the study. J.H.: one of the study designers, involved in the design and implementation of the surgical protocol. S.G.: the primary designer of the study, the surgical protocol.

## Conflicts of interest disclosure

The authors have no conflicts of interests.

## Research registration unique identifying number (UIN)

1. Name of the registry: Clinicaltrials.gov.
2. Unique Identifying number or registration ID: Panc-2023129.
3. Hyperlink to your specific registration (must be publicly accessible and will be checked): https://clinicaltrials.gov/ct2/show/NCT05723978

## Guarantor

Song Gao.

## Data availability statement

The dataset used and analyzed in this study were obtained from the corresponding authors upon reasonable request.

## Provenance and peer review

Not commissioned, externally peer-reviewed.

## Supplementary Material
