# Supplementary material for: Selective extended dissection for pancreaticoduodenectomy is associated with better survival in pancreatic cancer patients: retrospective cohort study
Source: Int J Surg. 2023 May 16;109(7):1852–62. doi: 10.1097/JS9.0000000000000437 (PMC10389680; doi:10.1097/JS9.0000000000000437)

## Supplementary

### Supplementary Table 1 Comparison of basic information between SD and SED group in original cohort

|  |  | Overall | SD | SED | p |
| --- | --- | --- | --- | --- | --- |
|  |  | 540 | 444 | 96 |  |
| Gender (%) | female | 244 (45.2) | 197 (44.4) | 47 (49.0) | 0.480 |
|  | male | 296 (54.8) | 247 (55.6) | 49 (51.0) |  |
| Age (%) | ≤60 | 268 (49.6) | 224 (50.5) | 44 (45.8) | 0.479 |
|  | >60 | 272 (50.4) | 220 (49.5) | 52 (54.2) |  |
| Tumor Size (%) | <=4 | 389 (72.0) | 323 (72.7) | 66 (68.8) | 0.505 |
|  | >4 | 151 (28.0) | 121 (27.3) | 30 (31.2) |  |
| AJCC Stage (%) | I/IIa | 232 (43.0) | 210 (47.3) | 22 (22.9) | <0.001 |
|  | IIb/III | 308 (57.0) | 234 (52.7) | 74 (77.1) |  |
| Positive LN ratio (%) | <=0.2 | 433 (80.2) | 361 (81.3) | 72 (75.0) | 0.206 |
|  | >0.2 | 107 (19.8) | 83 (18.7) | 24 (25.0) |  |
| Soft tissue invasion (%) | Negative | 114 (21.1) | 90 (20.3) | 24 (25.0) | 0.373 |
|  | Positive | 426 (78.9) | 354 (79.7) | 72 (75.0) |  |
| Vessel invasion (%) | Negative | 382 (70.7) | 298 (67.1) | 84 (87.5) | <0.001 |
|  | Positive | 158 (29.3) | 146 (32.9) | 12 (12.5) |  |
| Differentiation (%) | Poor | 236 (43.7) | 220 (49.5) | 16 (16.7) | <0.001 |
|  | Well | 304 (56.3) | 224 (50.5) | 80 (83.3) |  |
| CA19-9 (%) | ≤172.8 U/ml | 269 (49.8) | 215 (48.4) | 54 (56.2) | 0.201 |
|  | >172.8 U/ml | 271 (50.2) | 229 (51.6) | 42 (43.8) |  |
| CEA (%) | ≤3.54 ng/ml | 270 (50.0) | 224 (50.5) | 46 (47.9) | 0.736 |
|  | >3.54 ng/ml | 270 (50.0) | 220 (49.5) | 50 (52.1) |  |
| Nerve invasion (%) | Negative | 141 (26.1) | 115 (25.9) | 26 (27.1) | 0.912 |
|  | Positive | 399 (73.9) | 329 (74.1) | 70 (72.9) |  |
| iPNI (%) | iPNI (-) | 246 (45.6) | 206 (46.4) | 40 (41.7) | 0.465 |
|  | iPNI (+) | 294 (54.4) | 238 (53.6) | 56 (58.3) |  |
| Postoperative chemotherapy (%) | No | 144 (26.7) | 113 (25.5) | 31 (32.3) | 0.212 |
|  | Yes | 396 (73.3) | 331 (74.5) | 65 (67.7) |  |
| Curability (%) | R0 | 323 (69.1) | 254 (57.2) | 69 (71.9) | 0.008 |
|  | R1 | 217 (30.9) | 190 (42.8) | 27 (28.1) |  |

SD: Standard Dissection; SED: Selective Extended dissection; iPNI: imaging Perineural Invasion; CA19-9: carbohydrate antigen 19-9; CEA: carcinoembryonic antigen.

### Supplementary Table 2 Comparison of basic information between SED and RED group in original cohort

|  |  | SED | RED | p |
| --- | --- | --- | --- | --- |
|  |  | 96 | 92 |  |
| Gender (%) | Female | 47 (49.0) | 43 (46.7) | 0.874 |
|  | Male | 49 (51.0) | 49 (53.3) | |
| Age (%) | ≤60 | 44 (45.8) | 50 (54.3) | 0.307 |
|  | >60 | 52 (54.2) | 42 (45.7) | |
| Tumor Size (%) | <=4 | 66 (68.8) | 51 (55.4) | 0.083 |
|  | >4 | 30 (31.2) | 41 (44.6) | |
| P stage (%) | I/IIa | 22 (22.9) | 14 (15.2) | 0.248 |
|  | IIb/III | 74 (77.1) | 78 (84.8) | |
| LN ratio (%) | ≤0.11 | 48 (50.0) | 33 (35.9) | 0.071 |
|  | >0.11 | 48 (50.0) | 59 (64.1) | |
| Soft tissue invasion (%) | Negative | 24 (25.0) | 18 (19.6) | 0.472 |
|  | Positive | 72 (75.0) | 74 (80.4) | |
| Vessel invasion (%) | Negative | 84 (87.5) | 77 (83.7) | 0.592 |
|  | Positive | 12 (12.5) | 15 (16.3) | |
| Differentiation (%) | Poor | 16 (16.7) | 12 (13.0) | 0.622 |
|  | Well | 80 (83.3) | 80 (87.0) | |
| CA199 (%) | ≤181.5 U/ml | 54 (56.2) | 40 (43.5) | 0.109 |
|  | >181.5 U/ml | 42 (43.8) | 52 (56.5) | |
| CEA (%) | ≤3.48 ng/ml | 46 (47.9) | 48 (52.2) | 0.662 |
|  | >3.48 ng/ml | 50 (52.1) | 44 (47.8) | |
| Nerve invasion (%) | Negative | 26 (27.1) | 18 (19.6) | 0.296 |
|  | Positive | 70 (72.9) | 74 (80.4) | |
| iPNI (%) | iPNI(-) | 40 (41.7) | 32 (34.8) | 0.412 |
|  | iPNI(+) | 56 (58.3) | 60 (65.2) | |
| Postoperative chemotherapy (%) | No | 31 (32.3) | 65 (70.7) | <0.001 |
|  | Yes | 65 (67.7) | 27 (29.3) | |
| Curability (%) | R0 | 55 (57.3) | 50 (54.3) | 0.795 |
|  | R1 | 41 (42.7) | 42 (45.7) | |

SED: Selective Extended dissection; RED: routine extended dissection; iPNI: imaging Perineural Invasion; CA19-9: carbohydrate antigen 19-9; CEA: carcinoembryonic antigen.

### Supplementary Table 3 Perioperative complications were compared between SED and RED.

|  |  | Overall | SED | RED | p |
| --- | --- | --- | --- | --- | --- |
|  |  | 188 | 96 | 92 |  |
| Operative time (median [IQR]) |  | 296.73 [251.18, 331.86] | 282.29 [207.14, 333.46] | 306.88 [275.84, 330.87] | 0.007 |
|  | <=296.7 | 94 (50.0) | 54 (56.2) | 40 (43.5) | 0.109 |
|  | >296.7 | 94 (50.0) | 42 (43.8) | 52 (56.5) |  |
| Intraoperative bleeding (median [IQR]) |  | 420.00 [350.00, 502.50] | 415.00 [287.50, 532.50] | 420.00 [377.50, 490.00] | 0.586 |
|  | <=420 | 97 (51.6) | 50 (52.1) | 47 (51.1) | >0.999 |
|  | >420 | 91 (48.4) | 46 (47.9) | 45 (48.9) |  |
| Intraoperative transfusion (%) | No | 128 (68.1) | 65 (67.7) | 63 (68.5) | >0.999 |
|  | Yes | 60 (31.9) | 31 (32.3) | 29 (31.5) |  |
| combined devisceration (%) | No | 176 (93.6) | 91 (94.8) | 85 (92.4) | 0.708 |
|  | Yes | 12 (6.4) | 5 (5.2) | 7 (7.6) |  |
| Vessel recombination (%) | No | 173 (92.0) | 90 (93.8) | 83 (90.2) | 0.532 |
|  | Yes | 15 (8.0) | 6 (6.2) | 9 (9.8) |  |
| HLOS (mean (SD)) |  | 23.44 (8.55) | 19.43 (8.57) | 27.63 (6.22) | <0.001 |
|  | <=19 | 58 (30.9) | 52 (54.2) | 6 (6.5) | <0.001 |
|  | >19 | 130 (69.1) | 44 (45.8) | 86 (93.5) |  |
| Pancreatic fistula (%) | No | 110 (58.5) | 63 (65.6) | 47 (51.1) | 0.085 |
|  | Biochemical fistula | 65 (34.6) | 26 (27.1) | 39 (42.4) |  |
|  | Grade B | 13 (6.9) | 7 (7.3) | 6 (6.5) |  |
| Bile fistula (%) | No | 140 (74.5) | 72 (75.0) | 68 (73.9) | 0.997 |
|  | Yes | 48 (25.5) | 24 (25.0) | 24 (26.1) |  |
| Gastrointestinal fistula (%) | No | 176 (93.6) | 91 (94.8) | 85 (92.4) | 0.708 |
|  | Yes | 12 (6.4) | 5 (5.2) | 7 (7.6) |  |
| DGE (%) | No | 144 (76.6) | 84 (87.5) | 60 (65.2) | 0.001 |
|  | Yes | 44 (23.4) | 12 (12.5) | 32 (34.8) |  |
| Diarrhea (%) | No | 130 (69.1) | 74 (77.1) | 56 (60.9) | 0.025 |
|  | Yes | 58 (30.9) | 22 (22.9) | 36 (39.1) |  |
| Postoperative bleeding (%) | No | 169 (89.9) | 86 (89.6) | 83 (90.2) | >0.999 |
|  | Yes | 19 (10.1) | 10 (10.4) | 9 (9.8) |  |

SED: selective extended dissection, RED: routine extended dissection, IQR: interquartile range, HLOS: hospital length of stay, DGE: delayed gastric emptying

### Supplementary Table 4 Comparison of postoperative recurrence pattern between SD and SED

|  | Overall | SED | RED | p |
| --- | --- | --- | --- | --- |
|  | 188 | 96 | 92 |  |
| Residual pancreas (%) | 0 (0%) | 0(0%) | 0(0%) | NA |
| Liver metastasis (%) | 69 (36.7) | 27 (28.1) | 42 (45.7) | 0.019 |
| Lung metastasis (%) | 23 (12.2) | 11 (11.5) | 12 (13.0) | 0.913 |
| Bone metastasis (%) | 12 (6.4) | 5 (5.2) | 7 (7.6) | 0.708 |
| Peritoneal metastasis (%) | 16 (8.5) | 7 (7.3) | 9 (9.8) | 0.726 |
| Retroperitoneal (%) | 38 (20.2) | 19 (19.8) | 19 (20.7) | >0.999 |

SED: selective extended dissection; RED: routine extended dissection.

### Supplementary figure 1. The photos during surgery for the extent of extrapancreatic nerve plexus and lymph nodes of selective extended dissection (SED): A. type I extra-pancreatic perineural invasion (EPNI), B. type II EPNI and C. type III EPNI. The yellow region was the nerve plexus should be dissected and orange dots refer to the lymph nodes. (CHA: common hepatic artery, SA: spleen artery, SV: spleen vein, CT: celiac trunk, PLX I: extrapancreatic nerve plexus I, PLX II: extrapancreatic nerve plexus II, SMA: superior mesenteric artery, SMV: superior mesenteric vein)


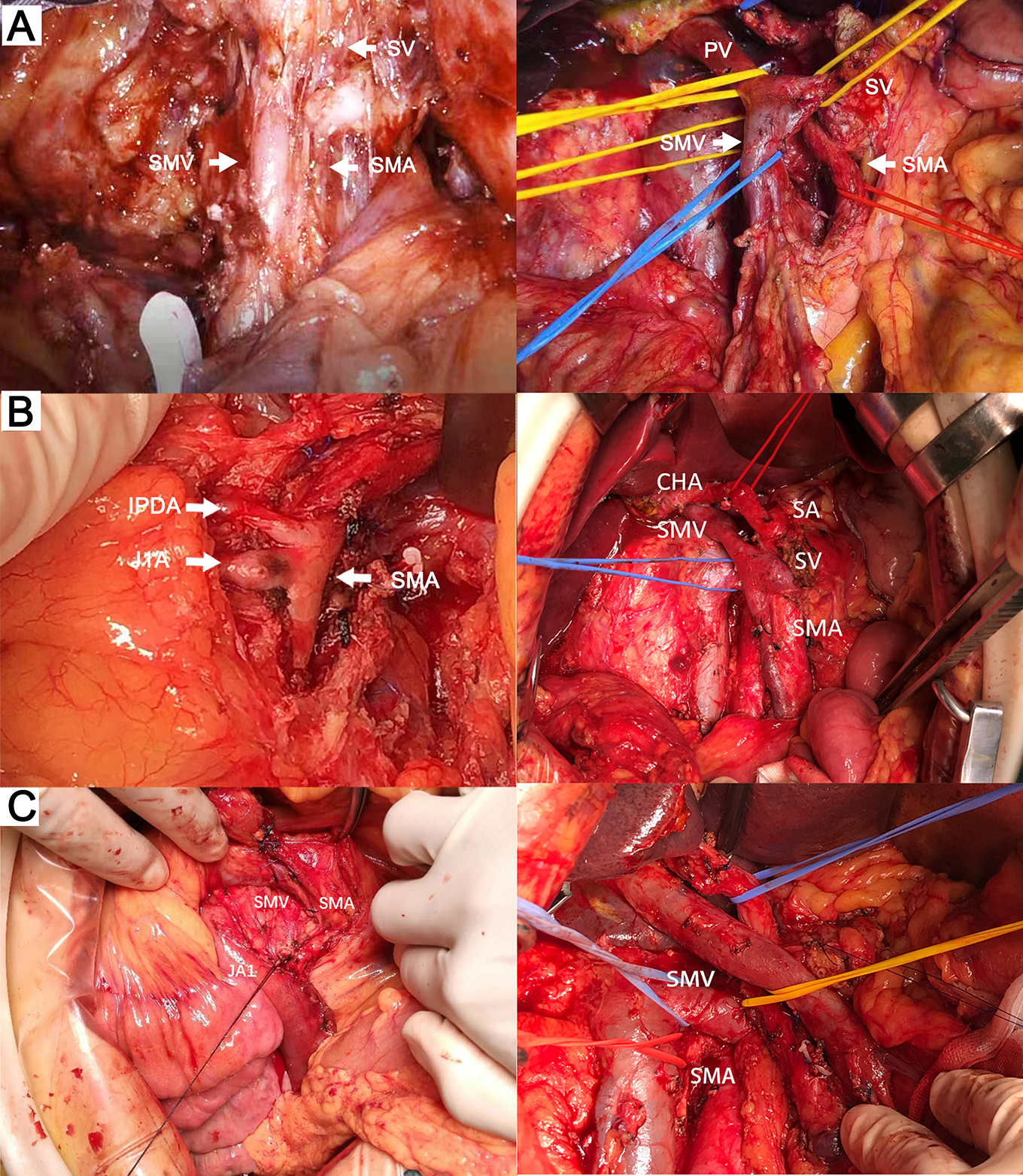


### Supplementary figure 2. Comparison of standard mean difference (SMD) between matched cohort and unmatched cohort.


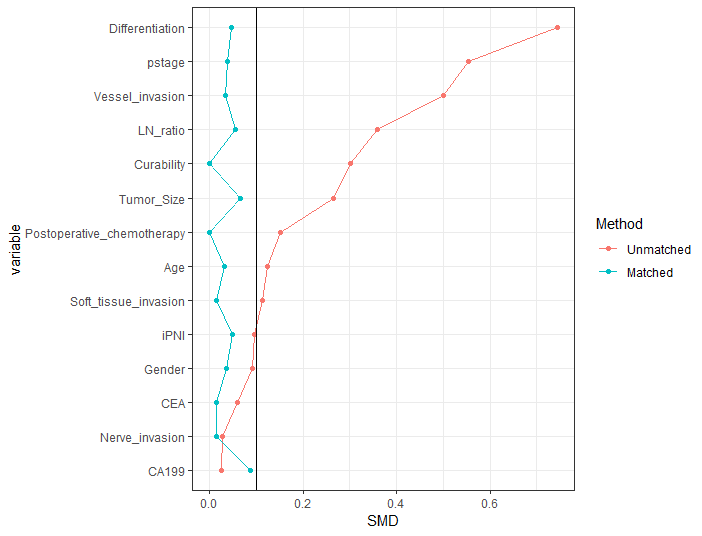


### Supplementary figure 3. K-M curve for patients underwent selective extended dissection (SED) and routine extended dissection (RED): A. in the total original cohort, B: in patients without extra-pancreatic perineural invasion (EPNI) and C: in patients with EPNI.


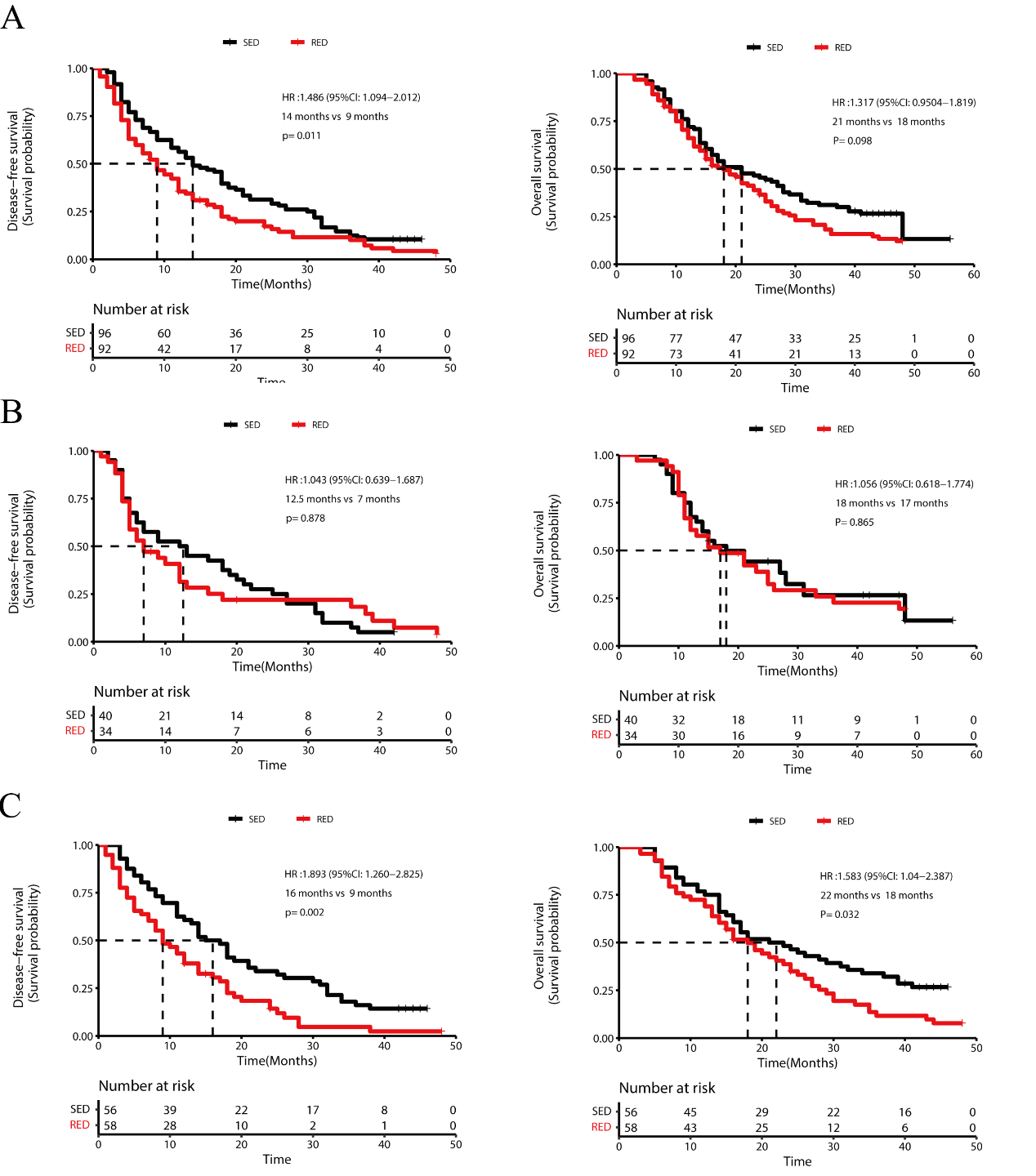

Supplement: Supplementary file 2 [file js9-109-1852-s002.docx]
